# Enhanced Charge Separation in Single Atom Cobalt Based Graphitic Carbon Nitride: Time Domain *Ab Initio* Analysis

**DOI:** 10.1021/acs.jpclett.3c03621

**Published:** 2024-02-19

**Authors:** Sraddha Agrawal, David Casanova, Dhara J. Trivedi, Oleg V. Prezhdo

**Affiliations:** † Department of Chemistry, University of Southern California, Los Angeles, California 90007, United States; ‡ Donostia International Physics Center (DIPC), 20018 Donostia, Euskadi, Spain; § IKERBASQUE, Basque Foundation for Science, 48009 Bilbao, Euskadi, Spain; ∥ Department of Physics, Clarkson University, Potsdam, New York 13699, United States; ⊥ Department of Physics and Astronomy, University of Southern California, Los Angeles, California 90007, United States

## Abstract

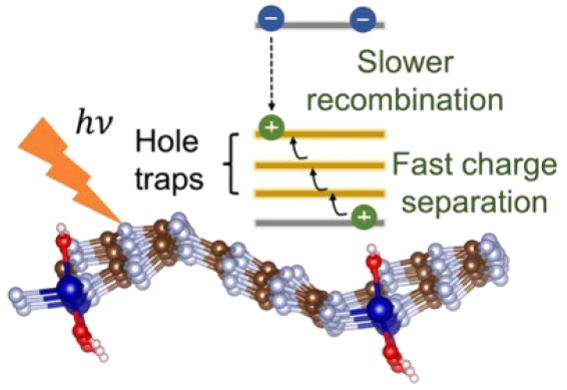

In recent years, single atom catalysts have been at the forefront of energy conversion research, particularly in the field of catalysis. Carbon nitrides offer great potential as hosts for stabilizing metal atoms due to their unique electronic structure. We use *ab initio* nonadiabatic molecular dynamics to study photoexcitation dynamics in single atom cobalt based graphitic carbon nitride. The results elucidate the positive effect of the doped cobalt atom on the electronic structure of GCN. Cobalt doping produces filled midgap states that serve as oxidation centers, advantageous for various redox reactions. The presence of midgap states enables the harvesting of longer wavelength photons, thereby extending the absorption range of solar light. Although doping accelerates charge relaxation overall, charge recombination is significantly slower than charge separation, creating beneficial conditions for catalysis applications. The simulations reveal the detailed microscopic mechanism underlying the improved performance of the doped system due to atomic defects and demonstrate an effective charge separation strategy to construct highly efficient and stable photocatalytic two-dimensional materials.

The use of carbon based materials for catalytic applications has garnered significant interest in recent years, owing to their tunable properties and low toxicity.^1^ Among these materials, graphitic carbon nitride (GCN) has emerged as a promising catalyst due to its stable, metal-free nature and ability to absorb visible light.^2−4^ However, the low surface area and poor charge transferability of GCN have hindered its widespread application in catalytic reactions.^5,6^ Various approaches have been explored to improve the catalytic properties of GCN, including doping with heteroatoms, creating defects, and incorporating metal nanoparticles.^7−10^ However, these methods often suffer from issues such as low stability, agglomeration of nanoparticles, and difficulty in controlling the size and dispersion of metal particles.

Single atom catalysts (SACs) have emerged as a new class of catalysts that can overcome some of the limitations of traditional metal nanoparticles.^11−13^ SACs consist of individual metal atoms dispersed on a support material, and their high catalytic activity and selectivity arise from the unique properties of isolated metal atoms. SACs exhibit excellent performance in many situations, including electrochemical, gas-phase, and liquid-phase reactions.^14^ The integration of SACs with GCN can improve the catalytic performance of GCN.^15−18^ The synthesis and characterization of SAC based GCN catalysts have been studied in recent years, with various metals being investigated for their suitability in this hybrid material.^19^ The successful integration of SACs with GCN has been demonstrated using metals such as Co, Pt, and Fe, among others.^20−23^ In these systems, the isolated metal atoms act as active sites for catalysis, improving the efficiency and selectivity of the GCN catalyst.

The catalytic activity of SAC based GCN catalysts has been evaluated for a wide range of reactions, including photocatalytic degradation of organic pollutants and electrocatalytic oxygen reduction reactions, such as CO_2_ reduction and selective oxidation of alcohols.^15,23−25^ In each of these applications, the SAC based catalysts exhibited superior performance compared with GCN without the inclusion of SACs. This is attributed to the enhanced charge transfer and catalytic activity of the isolated metal atoms in the hybrid material. The combination of SACs with GCN can potentially boost the catalytic properties of both materials by exploiting the synergistic effect between the isolated metal atoms and the GCN support.^16,18^

Herein, we report an excited state quantum dynamics study of nonradiative processes in cobalt single atom catalyst based GCN (Co-GCN) to gain insights into the nonequilibrium processes observed experimentally for photocatalysis. For this purpose, we employ a combination of *ab initio* time-dependent density functional theory (TDDFT) and nonadiabatic (NA) molecular dynamics (MD). The calculations demonstrate that Co-SACs can be stabilized on the surface of GCN through strong coordination bonds with the nitrogen atoms. We examine the effect of Co-SACs on the electronic properties of GCN and the efficiency of the resulting composite material for photocatalytic applications. The Co-SACs induce charge redistribution in the GCN framework, leading to the creation of new electronic states that enhance the catalytic activity of GCN. Our results indicate that Co centers can serve as oxidation centers and are beneficial for photoinduced charge separation. The calculations show that Co doping accelerates charge relaxation and recombination relative to that of pristine GCN. However, charge separation driven by the doping is fast, and charge recombination in Co-GCN is sufficiently slow to allow for the desired photocatalytic activity. The simulations provide a detailed mechanistic understanding of the atomistic origin of the improved performance of Co-GCN in various photocatalytic applications and offer guidance for the design of efficient materials for photocatalysis.

The simulations are performed using the mixed quantum–classical approach implementing NAMD within real-time TDDFT^26^ in the Kohn–Sham (KS) framework.^27,28^ The NAMD simulations are performed using the decoherence induced surface hopping (DISH) algorithm^29^ as implemented in the PYXAID software, under the classical path approximation.^30,31^ DISH incorporates the loss of coherence within the electronic subsystem induced by coupling to quantum vibrations.^32^ Decoherence effects become important in slow charge trapping and recombination processes taking place across large energy gaps.^33−36^ The decoherence time is estimated by computing the pure-dephasing time using the optical response theory formalism.^32,37,38^ A detailed mathematical description of the method can be found in previous papers.^30,31,39^ The method has been extensively applied to study excited state dynamics in a broad range of nanomaterials.^40−52^

Ground state geometry optimization, adiabatic MD, and NA coupling calculations are performed using the Vienna *Ab initio* Simulation Package (VASP),^53−55^ which uses a converged plane-wave basis. The Perdew–Burke–Ernzerhof (PBE) functional^56^ is used to calculate the exchange-correlation effects. To describe a strongly correlated system, a Hubbard *U* correction is applied with a value of *U*_eff_ = 3.0 eV for the Co 3d states.^57,58^ In general, although hybrid functionals, such as HSE06,^59,60^ are considered to be more reliable than PBE and PBE+*U*, DFT+*U* is still a popular method to treat strongly correlated systems at a lower computational cost. Because the +*U* correction is applied only to the Co atom and PBE tends to underestimate energy gaps, we employ the HSE06 functional as a reference to scale the PBE+*U* energy gaps. Specifically, we compute the electronic structure of the system in the optimized geometry using both the HSE06 and PBE+*U* functional and use the HSE06/PBE+*U* energy gap ratios to scale the PBE+*U* energy gaps obtained during MD simulations. Because the NA coupling is inversely proportional to the corresponding energy gap,^33^ we scale the NA couplings obtained by PBE+*U* by the inverse HSE06/PBE+*U* energy gap ratios as well.

The van der Waals interactions are described via the optB86b-vdW functional employed in the vdW-DF method.^61^ The plane-wave basis energy cutoff and the convergence criteria for energy and force are set to 750 eV, 10^–6^ eV/atom, and 0.01 eV/Å, respectively. A vacuum layer of 20 Å is added onto the Co-GCN surface to avoid the interaction between the layers in the *z*-direction. A 3 × 3 × 1 Γ-centered *k*-point mesh is used for the geometry optimization and adiabatic MD. A denser 9 × 9 × 1 *k*-mesh is further employed to obtain an accurate electronic structure with the PBE*+U* functional. VESTA software is used as a visualization tool.^62^ The NA coupling are calculated using the CA-NAC package.^63,64^ The NA couplings are obtained at the Γ-point because the system has a direct bandgap at the Γ-point.

The doped system is modeled using a 2 × 2 supercell with unit cell lattice parameters as given in ref (65). The system consists of 61 atoms which include 24 C, 32 N, 1 Co, 2 O, and 2 H atoms. After relaxing the geometry at 0 K, the system is heated to 300 K through repeated velocity rescaling for 2 ps. Then, 5 ps adiabatic MD trajectories are obtained in the microcanonical ensemble (NVE) with a 1 fs atomic time step. To specify the initial magnetic moment for each atom and the spin multiplet, MAGMOM and NUPDOWN = 3 parameters are used, improving the electronic structure convergence. The *ab initio* MD simulation demonstrates rapid fluctuations of electronic energy levels, well sampled by the 5 ps trajectory (Figure S1 of the Supporting Information). Therefore, 1 ns long NAMD simulations are performed by iterating the 5 ps NA Hamiltonian multiple times. The simulations are performed with the PYXAID software.^30,31^ To simulate the quantum dynamics of charge separation, trapping, and recombination, 100 initial configurations are selected randomly from the adiabatic MD trajectory, and 100 stochastic DISH sequences are sampled for each initial condition.

The optimized structure of Co-GCN is shown in Figure 1. A single Co atom is embedded in the center of a void of the GCN framework. The structure has been identified as the most stable for most transition-metal atoms including cobalt.^66^ Further, within this structure, Co is connected to two adjacent pyridinic N atoms of two distinct heptazine units and to two hydroxy (−OH) groups in order to mimic a stable four-coordinate complex.^67^

**Figure 1:**
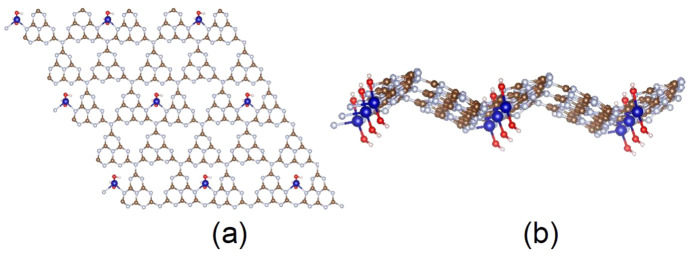
(a) Top and (b) side views of the optimized structure of cobalt–(OH)_2_ graphitic carbon nitride (Co-GCN). Atoms: C, brown; N, gray; Co, blue; O, red; H, pink. Cobalt with two OH groups on opposite sides of Co is introduced at the center of the GCN cavity (most stable site) to model a single atom catalyst based GCN. The cobalt atom is coordinated to two nitrogen atoms of the tri-*s*-triazine unit of GCN and as well as to the two hydroxy groups. The geometry is optimized after heating and is overall nonplanar.

Figure 2 shows the projected density of states (PDOS) of the system under investigation obtained by using two different functionals: PBE+*U* and HSE06. The PDOS is separated into contributions from C, N, Co, O, and H components. Here, we have shown the spin-polarized PDOS for the system because it has an odd number of electrons. Compared to the PBE+*U*, HSE06 opens up the bandgap between the valence band maximum (VBM) and conduction band minimum (CBM) and is closer to the experimental bandgap value of 2.7 eV.^2^ The Co doping introduces multiple localized electronic midgap states, exhibiting contributions from the hydroxy ligands. Based on the orbital occupancy, all of the defect states are completely filled and hence act as hole traps. This implies that the introduction of cobalt is beneficial for oxidation reactions, such as water oxidation, as one can utilize the trapped photoexcited hole on cobalt as an oxidation center. At the same time, the cobalt midgap states can promote charge recombination, generating additional relaxation pathways. Moreover, because the NA coupling is inversely proportional to the energy gap between states,^30,33^ the new relaxation pathways will be faster than the electron–hole recombination in undoped GCN. Thus, on the one hand, cobalt doping facilitates charge separation and creates a catalytic site, while on the other hand, it accelerates charge recombination. Therefore, it is important to evaluate the two effects in order to establish whether the benefits of charge separation outweigh the drawbacks of accelerated charge recombination.

**Figure 2:**
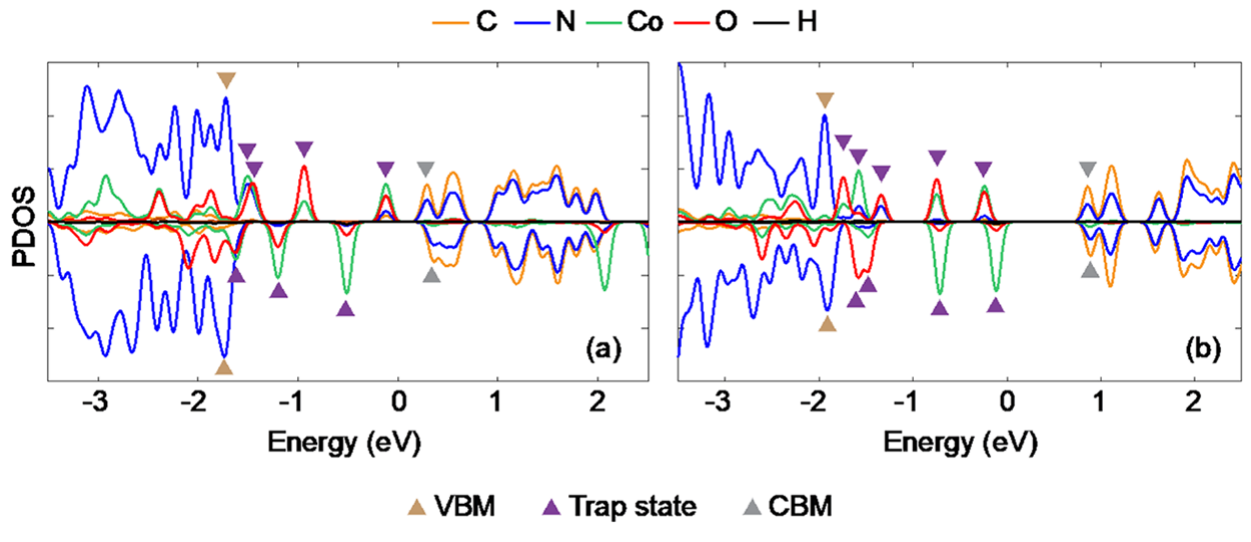
Spin-resolved atom projected density of states (PDOS) of Co-GCN obtained using (a) PBE+*U* and (b) HSE functionals. The positions of the band edge states are labeled as VBM and CBM, and the states between are trap states. The Fermi energy level is set to 0 in both cases. Energy gaps between the states obtained using HSE are used in the dynamics calculations because the bandgap obtained using the HSE functional is closer to the experimental bandgap of GCN.

Figure 3 shows spin-resolved charge densities of the band edge orbitals and trap states obtained using the HSE functional. The VBM and CBM charge densities are delocalized over large parts of the GCN framework and partly on the doped atoms. On the other hand, the charge densities of the midgap states are strongly localized on cobalt. Charge densities, obtained as squares of the corresponding wave functions, provide a visual representation that can be used to analyze the NA coupling strength. The NA coupling magnitude is closely related to the overlap between the charge densities of the two states. Localization of charge densities in different parts of the system leads to a decreased overlap and a smaller NA coupling.

**Figure 3:**
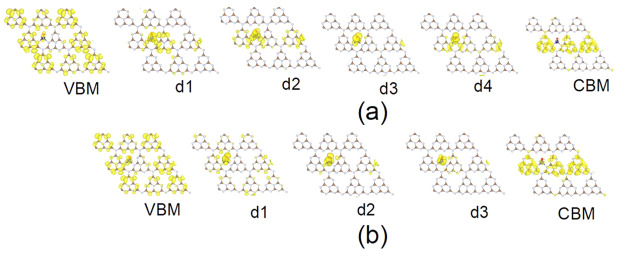
Charge densities (yellow) of the orbitals involved in the active space for (a) spin-up and (b) spin-down channels of Co-GCN obtained using the HSE functional. The charge densities for the band edge orbitals (CBM and VBM) are delocalized over the entire system, mostly excluding the defect region, while the charge densities for the defect trap states (d1–d4) are strongly localized near the doped Co atom.

Electron–vibrational interactions create inelastic and elastic electron–phonon scattering, and both types of scattering have a strong influence on charge trapping and recombination. Inelastic scattering, quantified by the NA coupling strength, leads directly to energy exchange between electrons and phonons during nonradiative relaxation. On the other hand, elastic scattering, characterized by the pure dephasing time,^32,37,38^ affects quantum coherence between initial and final states during a quantum transition and influences the transition indirectly.^33−36^ The charge trapping and recombination time scales are determined by NA coupling, energy gap, and pure dephasing time. In general, a larger energy gap, weaker NA coupling, and faster pure dephasing lead to slower dynamics.

**Table 1:** Canonically Averaged Energy Gaps and Absolute Nonadiabatic Couplings (NAC) between Pairs of States Involved in the Active Space for the Spin-Down Channel in Co-GCN^a^

orbitals	energy (eV)	scaled energy (eV)	NAC (meV)	scaled NAC (meV)
VBM–d1	0.09	0.11	42.08	34.93
d1–d2	0.42	0.76	24.31	13.37
d2–d3	0.69	0.60	14.44	16.61
d3–CBM	0.86	1.00	8.41	7.23
VBM–CBM	2.08	2.48	2.10	1.66

a The energy gaps and NAC are obtained using the PBE+*U* functional and are also scaled based on the HSE bandgap, as explained in the text.

The solar spectrum covers a broad range of energies, and in general, absorption of a photon places electrons and holes inside the bands away from the bandgap. However, relaxation of electrons and holes inside the bands through dense manifolds of states is fast (subpicoseconds) and is considerably faster than the charge trapping and recombination that take place across substantial energy gaps. Therefore, it is assumed in the simulations that the charges have already relaxed to the respective band edges. NAMD is then performed considering all possible electronic configurations in the active space constructed from the band edges and hole trap states, illustrated in Figure S2. In the current model, the nonradiative dynamics are simulated separately for the spin-up and spin-down channels. Evolution of populations of the multielectron states in each spin component is illustrated in Figure 4. The populations of all trap states are summed up together. More detailed data, including populations of each state separately, are presented in Figure S3. The corresponding time scales are reported in Tables 2 and S1. Analysis of the combined population of all trap states characterizes the time scales of charge separation and recombination, while the detailed information regarding population of each trap state provides data on the distribution of holes at different energies. Such information is important because the redox potential and efficiency of redox reactions are different for charges occupying different energy levels. The time scales reported in Tables 2 and S1 are obtained by fitting the relevant parts of the curve to exponential functions. Population decay is fitted to *P*(*t*) = *A* exp(−*t*/τ), and population rise is fitted to *P*(*t*) = *B*[1 – exp(−*t*/τ)]. The constants *A* and *B* are set to 1 for fitting the decay and rise of the populations of the excited and ground states, while these constants are treated as fitting parameters in the analysis of the trap states. To estimate the uncertainties in the reported time scales, we divided the entire 5 ps trajectory into five 1 ps parts and obtained individual time scales for each trajectory and then computed the standard deviations, as reported in Table 2. The time scales in the spin-up channel are relatively slower than those in the spin-down channel, and therefore the faster spin-down channel is considered for further analysis, as shorter time scales dominate the relaxation processes.

**Figure 4:**
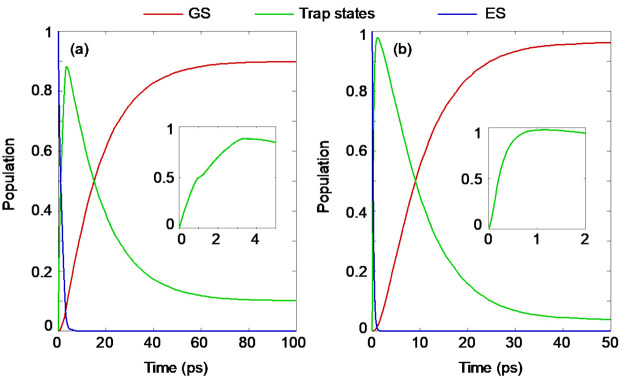
Nonradiative charge carrier trapping and recombination dynamics in (a) spin-up and (b) spin-down channels in Co-GCN. Insets show the fast rise of the trapped hole population of the respective spin channels. The corresponding time scales are given in Table 2. The trapped hole population is obtained by combining all of the individual trap state populations shown in Figure S3. The charge trapping time is significantly faster than the recombination time, which is beneficial for photocatalytic applications.

**Table 2:** Time Scales (ps) of Decay of Excited State (ES) Population, Rise of Trapped Hole Population, and Rise of Ground State (GS) Population, Corresponding to Figure 4^a^

	ES decay	trapped hole rise	GS rise
spin up	1.40 ± 0.22	1.43 ± 0.56	23.52 ± 2.90
spin down	0.32 ± 0.06	0.34 ± 0.08	12.18 ± 1.89

a Populations of all trap states are added to obtain the trapped hole population. Populations of individual trap states and corresponding time scales are shown in Figure S3 and Table S1.

Compared to pristine GCN,^39^ the Co-GCN system has multiple midgap trap states that provide additional pathways for nonradiative charge recombination, and as a result, the charge carrier lifetime is shorter in the doped system. This theoretical conclusion is in agreement with the experimental reports on shorter lifetimes of charge carriers in the single atom Co-doped GCN compared to the pristine GCN.^23,67,68^ The nonradiative relaxation becomes faster upon the doping due to the appearance of new relaxation channels with smaller energy gaps and stronger NA couplings. Although in the majority of applications one aims to achieve long-lived charge carriers, in the present case the drawback of the shortened carrier lifetime is outweighed by the benefit of the rapid charge separation and creation of the active catalytic sites by the cobalt doping. Further, the presence of midgap states extends the range of the absorbed light due to smaller energy gaps between occupied and empty states. In particular, the charge separation in the current system requires 0.3 ps in the faster spin-down channel, while the corresponding charge recombination takes 12 ps, more than a factor of 40 slower. The large energy gap and small NAC between d3 (hole) and CBM (electron) (Table 1) lead to slow electron–hole recombination, indicated by the rise of the GS population (Table 2 and Figure 4).

The ∼10 ps lifetime of the charge separated state is sufficient to initiate chemical reactions involving bond breaking and rearrangements. For example, the oscillation period of a typical chemical bond, such as C–O for CO_2_ reduction or O–H for H_2_O splitting, is shorter than 100 fs; i.e., the photocatalytic system has over 100 bond oscillation periods available to break a bond. Importantly, the current simulation cell is small due to computational limitations, restricting the charge carriers to be close to each other and making the recombination faster than in a real system. In large extended systems, charges can travel far from each other, and their recombination will be significantly slower.^69^ Both charge separation and recombination involve transitions between delocalized band states and localized trap states. As band states become more delocalized with increasing system size, both charge separation and recombination time scales will grow, but the separation will remain faster than the recombination. The experiments indicate that introduction of the dopant improves charge separation, while at the same time also shortens the carrier lifetime.^23,67,68^ The results reported here are in agreement with the experimental works, which demonstrate better efficiency of separation of photogenerated charge carriers and improved photocatalytic performance of single cobalt atom based GCN.^68,70^

In summary, we have studied the nonradiative charge separation and recombination dynamics in single atom cobalt-doped GCN by performing *ab initio* quantum dynamics simulations. The simulations demonstrate that introduction of cobalt produces occupied midgap states that serve as oxidation centers for redox reactions, in agreement with experimental results. The presence of midgap states increases the range of optical absorption in the solar spectrum by decreasing the energy gaps. Compared to the charge separation stemming from hole trapping, charge recombination is relatively slow due to larger energy gap and weaker NA coupling. Although the presence of trap states accelerates charge relaxation and recombination compared to the pristine system, as a result of additional recombination pathways, the overall photocatalytic performance of the doped system is better due to the enhanced charge separation rates. The performed simulations provide an atomistic understanding of the photoexcitation dynamics in a single atom catalyst based GCN and highlight its advantages in solar energy driven applications. The fundamental insights reported in this study assist in design of novel efficient materials for better photocatalytic applications.

## Supporting Information Available

Evolution of electronic energy levels, schematic of the electronic configurations in the active space, and detailed nonradiative relaxation dynamics data and corresponding time scales (PDF)
