# Supplementary material for: Enhanced Charge Separation in Single Atom Cobalt Based Graphitic Carbon Nitride: Time Domain Ab Initio Analysis
Source: J Phys Chem Lett. 2024 Feb 19;15(8):2202–8. doi: 10.1021/acs.jpclett.3c03621 (PMC10910588; doi:10.1021/acs.jpclett.3c03621)
Supplement: Supplementary file 1 — jz3c03621_si_001.pdf [file jz3c03621_si_001.pdf]

Supporting Information for

Enhanced Charge Separation in Single Atom Cobalt Based Graphitic Carbon Nitride: Time Domain Ab Initio Analysis

Sraddha Agrawal,[1] David Casanova,[2,3] Dhara J. Trivedi,[4,*] Oleg V. Prezhdo,[1,5]

[1] Department of Chemistry, University of Southern California, Los Angeles, California, USA

[2] Donostia International Physics Center (DIPC), Manuel Lardizabal Ibilbidea 4, 20018 Donostia, Euskadi, Spain

[3] Ikerbasque Foundation for Science, Plaza Euskadi 5, 48009 Bilbao, Euskadi, Spain

[4] Department of Physics, Clarkson University, Potsdam, New York, USA

[5] Department of Physics and Astronomy, University of Southern California, Los Angeles, California, USA

* Corresponding author. E-mail: dtrivedi@clarkson.edu

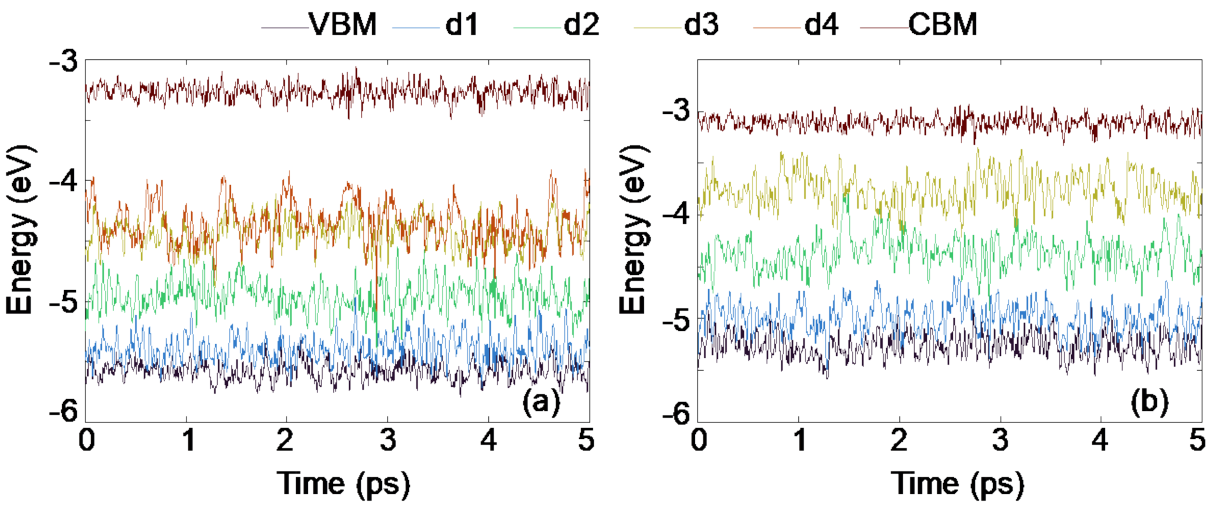

Figure S1. Energy levels for **(a)** spin up, and **(b)** spin down channels of Co-GCN. NVE calculations are done using the PBE+U functional and later used with appropriate scaling based on the HSE functional, as explained in the text, since the use of the HSE functional for MD is computationally excessively expensive.

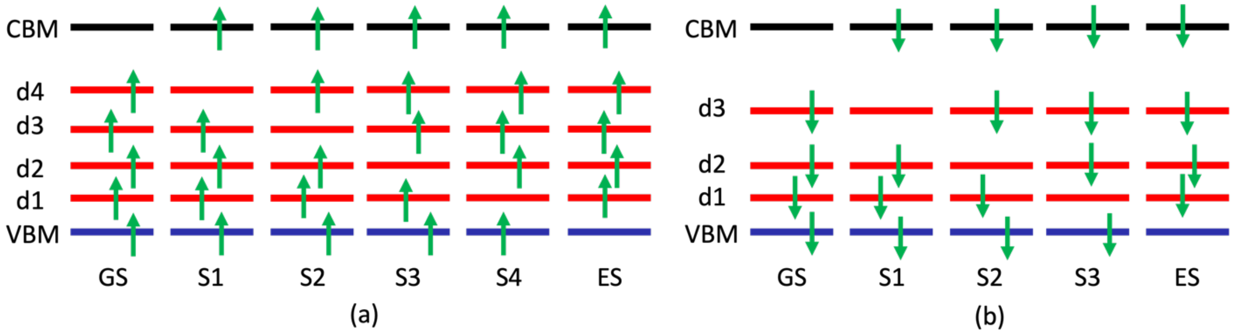

Figure S2. Schematic of the electronic configurations (NAMD basis functions) formed with the KS orbitals in the active space of the **(a)** spin up, and **(b)** spin down channels of Co-GCN. Color scheme: the VBM and CBM band edges are marked with blue and black lines, respectively, while the defect states are marked with red lines. Electrons are represented by green arrows.

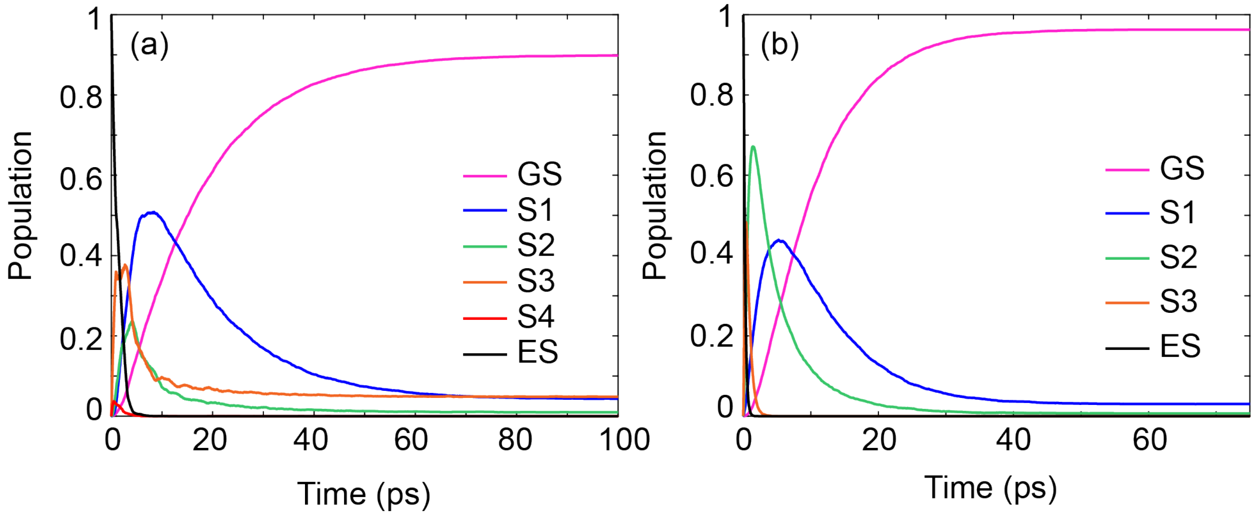

Figure S3. Non-radiative recombination dynamics in **(a)** spin up, and **(b)** spin down channels of Co-GCN. The plots are obtained by performing NAMD calculations after scaling the PBE+U energy and NAC values corresponding to the HSE energy values. The corresponding timescales are given in Table S1. The legends refer to the states shown in Figure S2.

Table S1. Non-radiative dynamics timescales (ps) for the states shown in Figure S3. The timescales are obtained by exponential fitting of the relevant parts of the plots. The rise of ground state gives the final recombination time of the photoexcited electron and hole. Timescales corresponding to the spin up configuration is relatively slower than the spin down channel.

	**ES decay**	**S1 rise**	**S2 rise**	**S3 rise**	**S4 rise**	**GS rise**
**Spin up**	1.40	4.15	2.51	0.46	0.28	23.52
**Spin down**	0.32	2.99	0.64	0.52	N/A	12.18
